# A simple peak detection and label-free quantitation algorithm for chromatography-mass spectrometry

**DOI:** 10.1186/s12859-014-0376-0

**Published:** 2014-11-25

**Authors:** Ken Aoshima, Kentaro Takahashi, Masayuki Ikawa, Takayuki Kimura, Mitsuru Fukuda, Satoshi Tanaka, Howell E Parry, Yuichiro Fujita, Akiyasu C Yoshizawa, Shin-ichi Utsunomiya, Shigeki Kajihara, Koichi Tanaka, Yoshiya Oda

**Affiliations:** Eisai Co., Ltd., Tsukuba, Ibaraki 300-2635 Japan; Shimadzu Corporation, Kyoto, 604-8511 Japan; iBio Tech Co., Ltd., Tsukuba, Ibaraki 305-0031 Japan

**Keywords:** Algorithm, Mass spectrometry, Label-free quantitation, Peak picking, Comparison

## Abstract

**Background:**

Label-free quantitation of mass spectrometric data is one of the simplest and least expensive methods for differential expression profiling of proteins and metabolites. The need for high accuracy and performance computational label-free quantitation methods is still high in the biomarker and drug discovery research field. However, recent most advanced types of LC-MS generate huge amounts of analytical data with high scan speed, high accuracy and resolution, which is often impossible to interpret manually. Moreover, there are still issues to be improved for recent label-free methods, such as how to reduce false positive/negatives of the candidate peaks, how to expand scalability and how to enhance and automate data processing. AB3D (A simple label-free quantitation algorithm for Biomarker Discovery in Diagnostics and Drug discovery using LC-MS) has addressed these issues and has the capability to perform label-free quantitation using MS1 for proteomics study.

**Results:**

We developed an algorithm called AB3D, a label free peak detection and quantitative algorithm using MS1 spectral data. To test our algorithm, practical applications of AB3D for LC-MS data sets were evaluated using 3 datasets. Comparisons were then carried out between widely used software tools such as MZmine 2, MSight, SuperHirn, OpenMS and our algorithm AB3D, using the same LC-MS datasets. All quantitative results were confirmed manually, and we found that AB3D could properly identify and quantify known peptides with fewer false positives and false negatives compared to four other existing software tools using either the standard peptide mixture or the real complex biological samples of *Bartonella quintana (*strain JK31). Moreover, AB3D showed the best reliability by comparing the variability between two technical replicates using a complex peptide mixture of HeLa and BSA samples. For performance, the AB3D algorithm is about 1.2 - 15 times faster than the four other existing software tools.

**Conclusions:**

AB3D is a simple and fast algorithm for label-free quantitation using MS1 mass spectrometry data for large scale LC-MS data analysis with higher true positive and reasonable false positive rates. Furthermore, AB3D demonstrated the best reproducibility and is about 1.2- 15 times faster than those of existing 4 software tools.

**Electronic supplementary material:**

The online version of this article (doi:10.1186/s12859-014-0376-0) contains supplementary material, which is available to authorized users.

## Background

Proteomics based on LC-MS is well established technology for discovery of disease biomarkers, drug target identification, mode of action (MOA) studies and safety marker identification in drug research. In particular, most of these analyses are differential, i.e., comparing samples from drug-treated and untreated subjects, diseased subjects and healthy controls, mutant and wild-type samples and so on. In cases of the differential analysis, mere identification of proteins and metabolites from the complex biological samples is not sufficient, and quantitative analysis is required. There are two main types of quantitation methods for differential analysis using LC-MS, one being the standard-free approach [1–4] and the other being the external/internal standards approach to normalize variations by the use of stable isotope labeling methods [5,6]. The latter approach offers high accuracy and reliability, but has the disadvantages of requiring expensive reagents and time-consuming preparation of standards in practical experiments. The former approach offers simplicity and easy experimental design with relatively low cost, but there remain many challenges related to bioinformatics, e.g., to increase true positives, to decrease false positives, to improve analysis performance for large scale data sets and to increase reliability by normalization of data. Meanwhile, proteomics data sets have become bigger and more complex in the last decade due to the increased sensitivity, resolution and throughput of LC-MS, thus to improve automated large-scale data handling is another challenge. In particular, it is also important to note that only 10-50% of spectra generated from LC-MS/MS have been correctly assigned for the identification in proteomics field [7]; this means that about half of the spectra, which were filtered during the identification process, may sometimes play important biological roles such as known and/or unknown post translational modifications. Therefore, label-free quantitation is a reliable, versatile and cost-effective method in the biomarker discovery field. To date, there are two major sub-approaches reported in label-free quantitation proteomics, i.e., 1) spectral counting based quantitation; 2) spectra intensity based quantitation. In this paper we will mainly focus on the latter one. Several label-free quantitative algorithms/tools are available for proteomics based on LC-MS data [8–10]. However, one of the current challenges is to develop a highly reliable and flexible LC-MS based quantitation method for large scale biomarker discovery. There are also only a few software tools which allow us to customize and/or add newly developed algorithms as plug-ins. Moreover, in most cases, existing algorithms were developed for only specific purposes, thus there are quite a few papers to date which compared different aspects of existing algorithms using the same data set. Here, we present a simple algorithm, which we call AB3D, which allows peak picking, isotope cluster recognition and quantitation using MS1 data with high reliability and with a sophisticated graphical user interface for verification using the universal mass spectrometry data visualization and analysis tool Mass++ [11], in addition to comparing different types of existing label-free quantitation algorithm.

### Algorithm

#### Data processing

**Figure 1:**
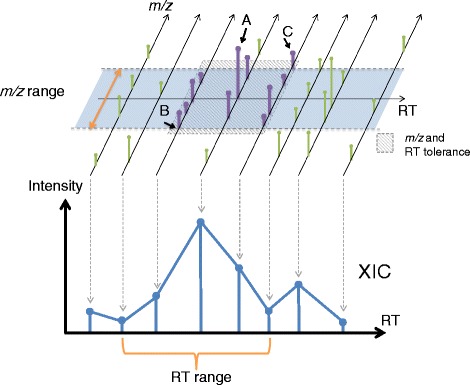
**A schematic overview of the AB3D peak detection algorithm.** AB3D sequentially processes all candidate peaks from the maximum intensities to minimum intensities. To extract the XIC (the bottom) for a target peak A (the upper), all related peak information such as *m/z*, retention time and intensity were obtained by searching adjacent peaks in the range of *m/z* and retention time tolerances. B and C are the minimum and maximum *m/z* values respectively within the tolerance.

In our laboratory, usually over a hundred megabyte raw data is generated per run by the most advanced LC-MS, thus it is important to considering high speed computational methods for data processing and spectra manipulation. In addition, to adapt the same algorithm for all the different types of mass spectrometry data format, it is useful to have a universal text format for internal pre-processing. AB3D first roughly picks all local maxima whose intensity is larger than a given threshold (for example, default 10000 for Orbitrap and 100 for QSTAR XL positive mode) as candidate peaks from the entire spectrum and then stores all the candidate peaks data into the memory, which consists of 3-dimensional mass spectrometry data, i.e., *m/z*, intensity and their corresponding retention time. Alternatively, all candidate peaks can be generated through an API (Application Programming Interface) which is provided from different mass spectrometry vendors such as MSFileReader (Thermo Fisher Scientific) and LCMSsolution(Shimadzu). AB3D generates extracted ion chromatograms (XICs) for all candidate peaks in the following steps (Figure 1);

1. Select the highest intensity peak A (target peak) among all the candidate peaks, then seek all the possible neighbour peaks within the range of *m/z* and retention time. If no neighbour peaks were found, then skip the second and third steps and move to the next highest intensity peak.
2. Determine the start data point B and end data point C *m/z* values for a XIC within a given *m/z* tolerance window.
3. Generate the XIC based on the ranges of *m/z* and retention time determined in step 2, and then the target peak and all its neighbour peaks will be removed from the candidate peaks.

### XIC Peak detection and quantitation

**Figure 2:**
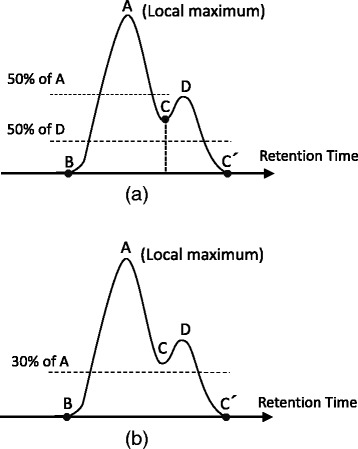
**The principle of the AB3D XIC peak detection algorithm.** For local maximum peaks A and D, B-A-C and C-D-C’ were picked as two candidate peaks when a horizontal line was drawn at 50% of the highest intensity **(a)**, while B-A-C’ were picked as one candidate XIC peak when a horizontal lines was drawn at 30% of the top intensity **(b)**.

In shotgun proteomics, many peaks may be buried in background noise and the complexity of the data is very high, therefore, it is difficult to assign all peaks. In addition, when samples are of very high complexity and the intensity of each peak is quite low, it was difficult to define the baseline in many cases. Thus, XIC peak detection is another challenge for performing highly reliable quantitative analysis. To address this, we have developed a novel mass chromatogram peak detection algorithm, in which we combine the local minimum and the weighted average peak detection algorithm to extract and compare peaks from highly complex data. Figure 2 shows the principle of our XIC peak detection algorithm. First, a point of highest intensity (apex A in Figure 2a) is found using a local maximum algorithm. Secondly, a check point at a given percentage (default 50%) of the peak height of the apex is used to discriminate the peak from noise, i.e., a horizontal line is drawn at the 50% height position along the RT axis, and if the line has two crossing points with the peak candidate, then the apex can be considered as a peak top. For calculating the peak area value, the start and the end points of the peak have to be determined, so next the lowest left and right points were found below the given percentage height position by using a local minimum algorithm, and these are considered to be the start (B in Figure 2a) and the end (C in Figure 2a) points, respectively. Finally, the peak area value can be calculated using these points, i.e., the start, apex, and end points. This algorithm is useful for analysis of complex samples, because the percentage from the peak top can be changed as required for particular experiments(Figure 2b); usually a higher percentage is used to detect the individual components of a complex XIC (like simple deconvolution) and a lower percentage is used to detect the intact complex XIC.

### XIC Peak filtering

In general, there are considerable pseudo-peaks originating from electronic and/or chemical noise for mass spectrometry-based quantitation [12], therefore, reduction of the noise peaks is another challenge for label-free quantitation. We have evaluated a large number of biological samples for optimizing a proper method of signal to noise ratio (S/N ratio) calculation and some useful filters for reducing false positive rate as follows.

S/N ratio:

1 $$ {P}_{baseline} = \left\{\begin{array}{c}\hfill {I}_{\frac{n+1}{2}}, \kern4.75em  if\ n\  is\  odd.\hfill \\ {}\hfill \frac{1}{2}\left({I}_{\frac{n}{2}}+{I}_{\left(\frac{n}{2}+1\right)}\right),\kern0.75em  if\ n\  is\  even.\hfill \end{array}\right. $$

2 $$ {P}_{noise} = \sqrt{\frac{{\left({I}_1-{P}_{baseline}\right)}^2+{\left({I}_2-{P}_{baseline}\right)}^2+\cdots +{\left({I}_n-{P}_{baseline}\right)}^2}{n}} $$

3 $$ {P}_{snratio} = \left({I}_1-{P}_{baseline}\right)\ /\ {P}_{noise} $$

where *n* is the total number of candidate peaks, *I* is the intensity value sorted in descending order as *I*_*1*_, *I*_*2*_, … *I*_*n*_. *P*_*baseline*_ is the baseline and *P*_*noise*_ is the noise level, respectively. As shown in formulas 1 and 2, the median value of intensities (*I*_*1*_ to *I*_*n*_ ) is used as the baseline and standard deviation is used as the noise level in AB3D.

Other major noise filters and isotopic clustering:

1. FWHM: full width at half maximum (FWHM) is well known as a noise reduction filter for spectra manipulation, it is also useful for reducing chemical and/or electrical noise for XIC peaks. The FWHM (default 0.05 min) can be adjusted according to the experimental condition and the instrument type.
2. Peak undulation: some ugly and bumpy XIC peak shapes might be generated due to insufficient data acquiring points, over loading of samples, low-spray status, and influence of overlapped other large peaks, we have focused on the number of up-downs within a XIC peak(we defined this as the peak undulation) and after processing and analysing over hundreds of biological samples, we found that peak undulation is one of the useful factors to reduce false positive peaks in most cases, the more numbers of peak undulation the lower data quality of XIC. Therefore we employed the peak undulation filter in AB3D as an adjustable parameter (default 3).
3. Number of spectra (data points) for each XIC peak: generally, XIC is constructed from multiple mass spectra and the number of spectra for each XIC peak can be considered as a factor to evaluate the quality of XIC peaks; a too small number of spectra for a XIC can be considered as a lack of confidence for advanced quantitation and identification, thus the number of spectra of the XIC filter is pre-set in AB3D.
4. Isotopic clustering: AB3D processes all sorted candidate peaks in descending order, first selects most intense peak as a target peak, then a *m/z* (1/charge) moving window is stepped backward and forward the target peak along *m/z* axis. If there is a peak signal within the moving window and the *m/z* tolerance, then the peak will be assigned as a member of an isotopic cluster and move to next, when there is no peak within the moving window and the *m/z* tolerance, the isotopic cluster is finally formed and a monoisotopic peak will be determined by using reported pattern matching method [13]. In order to address the overlapped peaks and the isotopic clusters, only the monoisotopic peak was removed from the candidate peaks before moving to the next target peak. The program ends when all the candidate peaks were assigned. In addition, one feature of our quantitative algorithm is that it has the ability to deal with highly complex proteomics data by merging ions that originate from the same molecule, even though they have different charge states and isotopes.

## Results and discussion

To evaluate the performance of the AB3D algorithm, comparison analyses were carried out by focusing on three aspects, i.e., 1) to evaluate the false positives and false negatives; 2) to evaluate the reliability of quantitation results; 3) to evaluate the execution time for each algorithm. While target software for comparison, were carefully selected using the following criteria, which have a similar concept as AB3D; 1) label-free quantitation using MS1; 2) widely used and freely available; 3) run on Windows OS, therefore, MZmine 2 [14], MSight [15], SuperHirn [16] and OpenMS [17] were chosen finally for comparison in this study. MZmine 2 is Java-based label-free software package using MS1 data for quantitation and has capabilities for GUI and batch based multi-runs. MSight is a peak detection algorithm based on methodology for handling and analysing two dimensional gel image data. SuperHirn is a C++ program with capabilities for alignment of all LC-MS runs as well as peak picking and quantitation. OpenMS is a C++ library based open source software which provides functionalities for handling and analysing proteomics data. All the software packages (MZmine 2, MSight, SuperHirn and OpenMS) were downloaded from their recommended site and run locally.

### Peak detection results comparison

To assess the performance of AB3D and the previous algorithms, two data sets which acquired from different types of instruments, were prepared (data set 1 and data set 2). For data set 1, we conducted a LC-MS experiment using a standard peptide mixture consisting of BSA peptides with different concentrations and peptides derived from four standard proteins such as beta galactosidase, phosphorylase b, myoglobin and cytochrome c with the same concentration (see details in the Method section). The mass spectra were collected using LTQ-orbitrap (Thermo Fisher Scientific) mass spectrometer and four technical replicates of prepared samples were conducted for each BSA peptide concentration and all acquired raw MS1 and MS/MS data were input into all the peak detection algorithms (AB3D, MZmine 2, MSight, SuperHirn and OpenMS) to produce peak lists for each peptide spectrum. Peaks identified as peptides of BSA or four standard proteins by either Mascot [18] or X! Tandem [19] from multiple samples and with higher peptide scores (>25) for Mascot and the expectation value <0.1 for X!Tandem, were set as true positive (TP). Peaks which were not identified as peptides from known BSA or the other four standard proteins were considered as false positive (FP) in this study. Parameter settings for each software package are provided in Additional file 1. All identification results using Mascot and X!Tandem and features detected by each of all five algorithms are provided in Additional file 2 and Additional file 3, respectively.

Furthermore, to examine our algorithm can adapt for other instruments as well, a real biological dataset was obtained from the PRIDE database [20] as data set 2, in which Fabietti et al reported an extensive shotgun proteomic analysis of *Bartonella quintana*, and the mass spectra were acquired using QSTAR XL (AB Sciex) for Oklahoma and JK31 strains [21]. We used JK31 stain raw data which contains 5 fractionations and 3 replicates for each fractionation in total 14 raw files (one missing replicate in fraction 4). All identification results were obtained from supporting information provided at the publisher’s web-site (see details in the Methods section), and the peptides/proteins identified in the literature were set as TP in our study. Raw (.wiff) data was converted to mzXML format files by using proteoWizard [22], then the peak lists were produced using AB3D, MZMine 2, MSight, SuperHirn and OpenMS algorithms, respectively. Parameter tuning was conducted 9-26 times for each algorithm and the best result was used for comparison finally. The different times of parameter tuning are due to the different number of parameters for each algorithm. We focused on the critical parameters, and tuned as many patterns as possible for each algorithm. The tuning parameters for each algorithm are provided in Additional file 4.

Table 1 summarises peak picking results generated by each software package for BSA standard peptide concentrations from 96 amol to 300 fmol with mixed standard peptides from four proteins. The details about peptide identification and parameters for all peak lists generated by the five algorithms using data set 1 are described in Methods. Similarly, Table 2 shows peak picking results produced by each algorithm for data set 2. Results shown in Tables 1 and 2, demonstrate that AB3D has the least number of total generated peaks but has relatively higher TP while keeping lower FP comparing with the other four existing algorithms for data sets 1 and 2. The total numbers of unique peptides identified from all replicates were also listed for each data set; clearly AB3D has the distinction of relatively higher numbers of TP while keeping reasonable false positives compared with other software algorithms. Furthermore, TP and FP rates of the unique peptides were calculated (formulas 4 and 5) from each peak list and scatter plots were obtained for comparison.

**Table 1:** **Summary of the numbers of peaks generated by AB3D, MZmine 2, MSight, SuperHirn and OpenMS for standard peptide mixture consisting of BSA peptides with different concentrations (96 amol, 480 amol, 2400 amol, 12 fmol, 60 fmol and 300 fmol) and peptides derived from four standard proteins such as beta galactosidase, phosphorylase b, myoglobin and cytochrome c with the same concentrations, and their replicates (n = 4) using Data set 1**

**BSA Conc.**	**N**	**AB3D**			**MZmine2**			**MSight**			**SuperHirn**			**OpenMS**		
		**Total**	**TP**	**FP**	**Total**	**TP**	**FP**	**Total**	**TP**	**FP**	**Total**	**TP**	**FP**	**Total**	**TP**	**FP**
300 fmol	1	1,482	208	1,274	4,980	240	4,740	1,317	169	1,148	2,009	201	1,808	3,305	240	3,065
	2	1,458	205	1,253	4,901	246	4,655	1,155	175	980	1,896	202	1,694	3,393	240	3,153
	3	1,452	207	1,245	5,047	235	4,812	1,437	176	1,261	2,007	201	1,806	3,214	239	2,975
	4	1,415	214	1,201	4,875	239	4,636	1,333	173	1,160	1,919	205	1,714	3,052	228	2,824
	UKP		261			276			195			249			263	
60 fmol	1	1,022	189	833	2,620	193	2,427	1,062	164	898	1,182	167	1,015	1,340	198	1,142
	2	1,033	185	848	2,521	185	2,336	1,047	166	881	1,257	173	1,084	1,264	193	1,071
	3	975	181	794	2,374	187	2,187	909	152	757	1,160	165	995	1,239	190	1,049
	4	950	181	769	2,318	186	2,132	833	154	679	1,187	167	1,020	1,153	185	968
	UKP		217			220			177			202			210	
12 fmol	1	752	145	607	1,474	146	1,328	603	128	475	869	130	739	773	150	623
	2	742	148	594	1,449	150	1,299	513	121	392	858	140	718	754	147	607
	3	695	151	544	1,262	145	1,117	624	128	496	789	128	661	680	148	532
	4	673	154	519	1,234	143	1,091	518	122	396	752	130	622	642	141	501
	UKP		177			170			134			170			161	
2400 amol	1	655	122	533	1,279	124	1,155	440	98	342	747	107	640	619	118	501
	2	653	115	538	1,193	113	1,080	414	93	321	769	107	662	585	118	467
	3	598	117	481	1,112	113	999	360	86	274	716	105	611	581	117	464
	4	612	118	494	1,080	116	964	368	87	281	689	112	577	553	114	439
	UKP		138			135			100			137			128	
480 amol	1	668	101	567	1,237	108	1,129	450	83	367	778	87	691	630	100	530
	2	622	94	528	1,191	97	1,094	431	81	350	768	89	679	592	94	498
	3	608	100	508	1,094	103	991	388	76	312	731	99	632	569	90	479
	4	593	93	500	1,063	93	970	358	71	287	688	94	594	498	93	405
	UKP		115			123			88			118			104	
96 amol	1	688	97	591	1,488	102	1,386	433	76	357	730	83	647	673	89	584
	2	636	88	548	1,311	92	1,219	408	71	337	717	83	634	658	87	571
	3	583	92	491	1,103	89	1,014	379	68	311	703	84	619	584	77	507
	4	553	88	465	1,060	87	973	316	64	252	697	85	612	489	76	413
	UKP		106			110			79			110			94	

Values in individual rows for each concentration represent the individual number of known peptides of BSA and the four other standard proteins from 4 replicates. Values in UKP rows represent the total numbers of unique known peptides identified from 4 replicates. TP and FP represent true positive and false positive, respectively.

**Table 2:** **Summary of the numbers of peaks generated by AB3D, MZmine 2, MSight, SuperHirn and OpenMS for the real biological data (data set 2)**

**Fraction.**	**N**	**AB3D**			**MZmine2**			**MSight**			**SuperHirn**			**OpenMS**		
		**Total**	**TP**	**FP**	**Total**	**TP**	**FP**	**Total**	**TP**	**FP**	**Total**	**TP**	**FP**	**Total**	**TP**	**FP**
FK1	1	496	197	299	529	155	374	328	147	181	760	105	655	500	195	305
	2	498	193	305	469	148	321	365	172	193	739	112	627	447	190	257
	3	490	211	279	503	150	353	379	178	201	808	127	681	484	205	279
	UKP		279			231			234			209			258	
FK2	1	1,049	426	623	1,117	344	773	779	392	387	1,416	254	1,162	917	390	527
	2	1,074	433	641	1,087	337	750	743	386	357	1,517	248	1,269	813	352	461
	3	1,416	515	901	1,454	404	1,050	767	429	338	2,036	333	1,703	1,284	440	844
	UKP		651			547			547			496			551	
FK3	1	613	330	283	651	275	376	686	323	363	763	184	579	528	310	218
	2	725	344	381	757	280	477	675	310	365	975	194	781	636	349	287
	3	657	372	285	897	373	524	754	298	456	1,064	236	828	604	344	260
	UKP		489			468			481			366			453	
FK4	2	1,476	446	1,030	1,465	335	1,130	1,317	430	887	1,969	266	1,703	1,064	355	709
	3	1,452	576	876	1,705	509	1,196	1,574	533	1,041	2,064	361	1,703	1,213	506	707
	UKP		681			597			650			474			566	
FK5	1	1,709	423	1,286	1,674	329	1,345	526	251	275	2,128	237	1,891	643	229	414
	2	2,038	425	1,613	2,231	349	1,882	721	305	416	2,988	259	2,729	834	274	560
	3	2,031	572	1,459	2,432	509	1,923	1,696	489	1,207	3,126	340	2,786	1,186	401	785
	UKP		768			687			636			553			516	

Values in individual rows for each fraction represent the individual numbers of identified peptides from 3 replicates. In fraction 4, results for two replicates were presented because there was one missing replicate. Values in UKP rows represent the total numbers of unique known peptides identified from 2-3 replicates. TP and FP represent true positive and false positive, respectively.

4 $$ TPR = \frac{\mathrm{x}}{t\ } $$

5 $$ FPR = \frac{\mathrm{y}-\mathrm{x}}{\mathrm{z}} $$

where *TPR* is the TP rate, *FPR* is the FP rate, *x* is the number of unique peaks identified as known peptides from all replicates for data set 1 and 2, *t* is the total number of unique peptides identified as from BSA and the four standard proteins for data set 1 (*t* =293, Table 3) or the total number of identified unique peptides in each fraction for data set 2. *y* is the number of peaks detected for each individual MS data file and *z* is the average peaks detected from each concentration of data set 1 and each fraction of data set 2, respectively.

**Table 3:** **Summary of BSA and four other standard proteins used in Data set 1**

	**Length**	**Peptides**	**Peaks**	**Coverage(%)**
BSA	607	193	210	87.15
Beta galactosidase	1,023	45	50	34.80
Phosphorylase B	842	33	34	32.30
Myoglobin	153	11	11	50.98
Cytochrome C	104	11	11	60.58
Total (peptides, peaks)		293	316	

Length is the total sequence length of each standard protein. Peptides is the number of peptides identified in this experiment. Peaks is the total number of peaks observed and Coverage (%) is the coverage for identified peptides.

**Figure 3:**
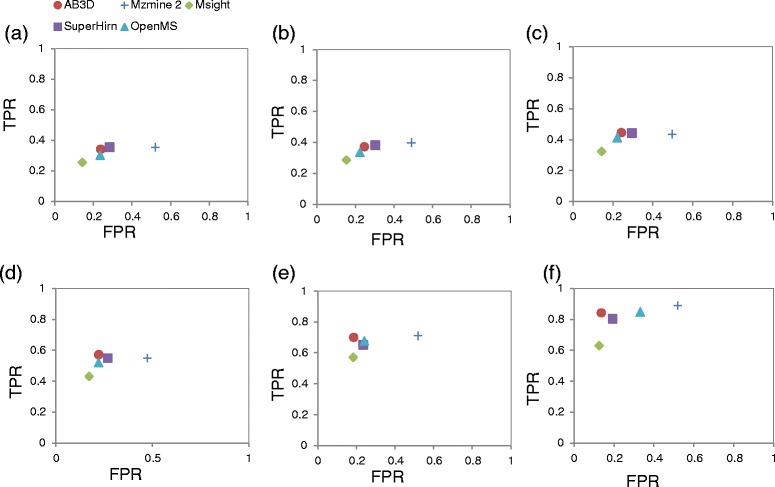
**Scatter plots of FPR and TPR for unique known peptides identified from four replicates for each algorithm.** Standard peptide mixture with BSA concentration **(a)** 96 amol, **(b)** 480 amol, **(c)** 2400 amol, **(d)** 12 fmol, **(e)** 60 fmol and **(f)** 300 fmol. AB3D (filled circle ), MZmine 2 (plus), MSight (filled diamond), SuperHirn (filled rectangle), and OpenMS (filled triangle). The same datasets (data set 1) were used to generate peak lists from those five algorithms. FPR for unique known peptides are calculated using the average of each replicate FPR.

**Figure 4:**
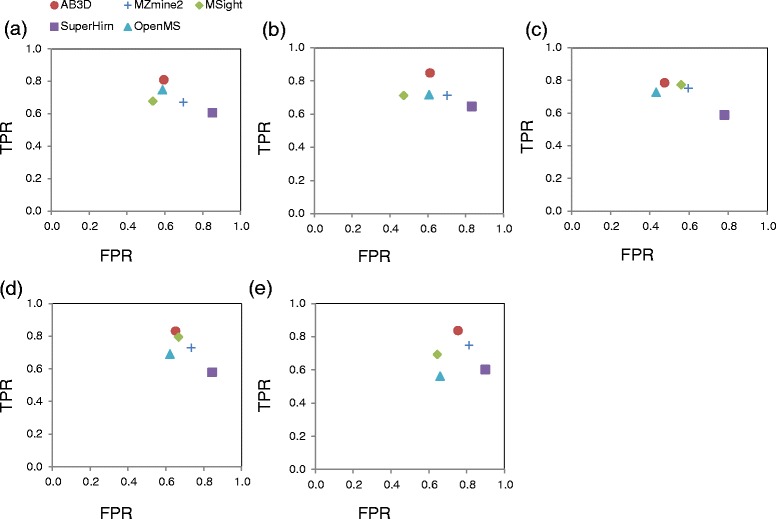
**Scatter plots of FPR and TPR for unique peptides identified from three replicates for each algorithm using data set 2. (a)** fraction 1, **(b)** fraction 2, **(c)** fraction 3, **(d)** fraction 4 and **(e)** fraction5. AB3D (filled circle), MZmine 2 (plus), MSight (filled diamond), SuperHirn (filled rectangle) and OpenMS (filled triangle). The same datasets (data set 2) were used to generate peak lists from those five algorithms. FPR for unique peptides are calculated using the average of each replicate FPR.

Figure 3(a) to (f) illustrate scatter plots of *TPR* against *FPR* of the unique known peptides shown in Table 1 for BSA standard peptide concentrations from 96 amol to 300 fmol with mixed standard peptides from four proteins. Similarly, Figure 4(a) to (e) shows the scatter plots of *TPR* against *FPR* of the unique peptides shown in Table 2 for data set 2.

In general for a ROC plot, the closer to the top left the better for performance, by comparing the known unique peptides shown in Figure 3(a) to (f), AB3D has a better balance of *TPR* and *FPR*, i.e., AB3D is mostly located on the upper left, shows the higher true positive rate while keeping lower false positive rate in all BSA peptide concentrations. Figure 4 shows similar results as those shown in Figure 3 even for the real complex biological samples (data set 2), which demonstrated that AB3D has the better performance for label-free quantitation using mass spectrometry data.

### Quantitation and performance comparison

**Figure 5:**
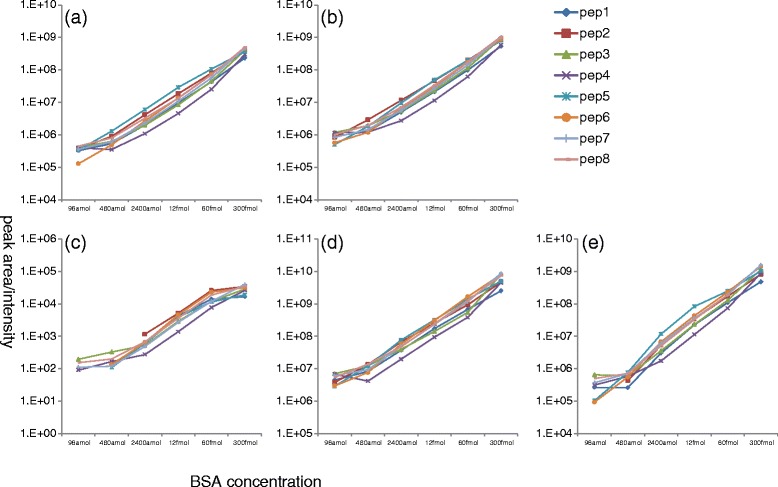
**Correlations between peak quantitative values of (a) AB3D, (b) MZMine 2, (c) MSight, (d) SuperHirn and (e) OpenMS** ***vs*** **the BSA digestion standard concentration (from 96 amol to 300 fmol) for 8 peptides using Data set 1.** Pep1, pep2, pep3, pep4, pep5, pep6, pep7 and pep8 represented FKDLGEEHFK, YLYEIAR, SLHTLFGDELCK, RHPEYAVSVLLR, TCVADESHAGCEK, LKECCDKPLLEK, KVPQVSTPTLVEVSR and LVNELTEFAK peptides of BSA, respectively.

**Figure 6:**
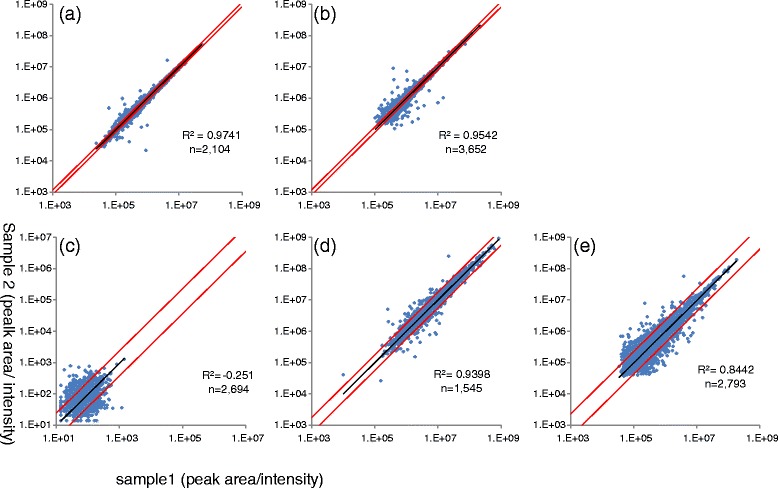
**The scatter plot of peak picking and quantitative results for each algorithm using Data set 3.** The total number of detected peaks are 2104, 3652, 2694, 1545, 2793 and the correlation coefficients between two replicates are R^2^ = 0.9741, 0.9542, -0.251, 0.9398, 0.8442 for **a** (AB3D), **b** (MZmine 2), **c** (MSight), **d** (SuperHirn) and **e** (OpenMS), respectively. 2SD (red lines) was also plotted for each algorithm.

**Figure 7:**
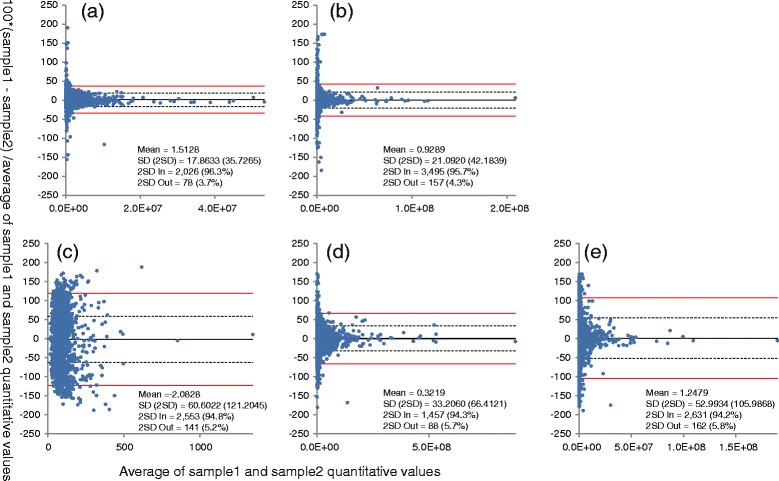
**The Bland-Altman plot for peak picking and quantitation results utilizing a complex peptide mixture of HeLa cell proteins digested peptides and 96 amol concentration BSA digestion standard (Data set 3) with 1 (dotted line) and 2 (red line) SD of mean (black line) for a (AB3D), b (MZmine 2), c (MSight), d (SuperHirn) and e (OpenMS), respectively.** 2SD In represented the number of peaks which fall within 2SD, and 2SD Out represented the number of peaks which fall outside of 2SD, respectively.

To test the quantitative accuracy of AB3D, correlation analysis was carried out by plotting peak area *vs* BSA peptide concentration for 8 identified BSA peptides which have no or fewer missing peak area values for all BSA peptide concentrations (data set 1) from 96 amol to 300 fmol and the results shown in Figure 5(a) to (e). Overall the results indicated that there are good positive correlations between quantitative value (peak area) and BSA peptide concentrations for all 5 algorithms, while AB3D and SuperHirn show better linearity than the others from low to high BSA concentrations. Some peptides showed less linearity at low concentration ranges, which was considered to be due to peptide adsorption on the sample plate. Furthermore, to assess reliability of AB3D and the other algorithms in their practical case for differential analysis, mixture samples consisting of peptides prepared from HeLa cells and BSA standard peptides with concentration of 96 amol, were prepared as a third data set (data set 3) for this study. The differences of peak picking and quantitative results for two technical replicates were then evaluated by the scatter and Bland-Altman plot [23] (Figures 6 and 7). In Figures 6 and 7, peak area values were used for AB3D, MZmine 2 and MSight, volume values were used for SuperHirn and intensity values were used for OpenMS, which does not provide area values. Parameters and detected features (RT and *m/z*) using data set 3 for each software package are provided in Additional file 1 and Additional file 5, respectively. Moreover, after analysing RT drift of BSA peptides between two replicates, peak matching tolerances for *m/z* and RT between two replicates are set as 0.01 Da and 0.2 min, respectively. In general, for Figure 6, the closer to the y = x line the better reproducibility for each algorithm. As shown in Figure 6, AB3D showed the smallest range of 2SD and the best correlation between two replicates with a correlation coefficient of R^2^ = 0.97 comparing with the other four algorithms. The corresponding Bland-Altman plots for Figure 6 were produced as shown in Figure 7, where the mean value is represented as a percentage of average difference, the range of 2SD is represented as the ability to reduce the ambiguous results and peaks located outside of 2SD (called outliers hereafter) considered as there are significant differences between two replicates, respectively. As shown in Figure 7, clearly the AB3D algorithm has the smallest range of 2SD with a value of 17.86%, and the smallest outliers with a value of 3.7% comparing with the other four algorithms. These results demonstrated that AB3D has a higher potential to reduce the false positive peaks and find the true changed analytes in differential analysis even using complex biological samples.

**Figure 8:**
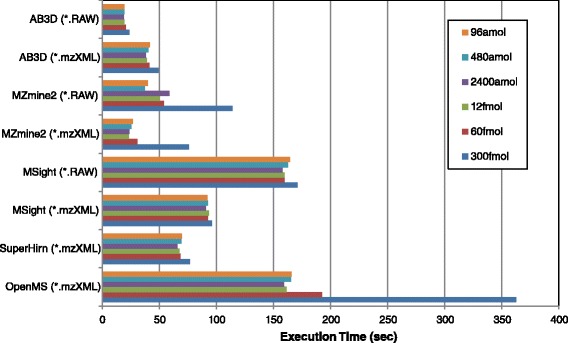
**Comparison of the execution time (sec) for peak detection and quantitation using AB3D, MZmine 2, MSight, SuperHirn and OpenMS, respectively.** The same data (data set 1) and workstation were used for all five algorithms and the workstation specification is shown as following: OS: Windows 7 Professional (64bit) Service Pack 1; CPU: Intel Xeon E5520 2.27 GHz 2.26 GHz (2 Processor); RAM: 8.00 GB.

To evaluate the computational performance of the AB3D algorithm, execution time analysis was conducted for comparison. All five algorithms used the same raw files (data set 1) and computer in the same conditions, execution times for each algorithm were measured from data input to finishing peak detection and their corresponding quantitative values; note the MS data format conversion time was not included in the measured time. For a LC-MS file, triplicate measurements were carried out and the average execution values for each algorithm were used for comparison. Since SuperHirn only accept mzXML format, therefore we performed execution time comparison by using mzXML files firstly although currently the recommended standard format is mzML; however, if the software can also handle raw format, execution times for raw input files were also measured and listed up for comparison, and we selected the best one for software which has more than two executing time values for final evaluation. Figure 8 shows the results for these five algorithms, and it demonstrates that the execution time for AB3D is about 1.2 to 15 times faster compared with MZmine 2, MSight, SuperHirn and OpenMS. This is one of the motivations for using AB3D to save the computational time and to allow research efficiently handling bigger and complex data sets due to either the increased sensitivity, resolution, throughput of LC-MS or the increase of biological sample numbers. AB3D is a 2D based peak picking algorithm and faithfully detects 2D peaks from largest to smallest in descending order. Moreover, the plug-in style development and the fewer model fitting approaches are the other major features that need to be highlighted. In contrast, MZMine 2, OpenMS are using different types of models for fitting the peaks and MSight generates images from the raw data file for adapting the image-based peak detection, which would be possible reasons for relatively expensive computational tasks. SuperHirn uses fewer model fitting algorithms but only accept mzXML format. Recently, MaxQuant [24] algorithm was developed by using 3D features, and it was reported as a more effective way of peak picking but it was optimized for Orbitrap and mainly focusing on SILAC based quantitation although it has label-free functionality also, therefore MaxQuant was not used as a benchmark candidate in this study.

In general, the more functionality the more complicated the software operation, in AB3D, only 3 steps are needed to perform peak detection and quantitation, i.e., 1) read MS file; 2) optimize AB3D parameters using a heatmap; and 3) process peak detection and quantitation. These results demonstrated that AB3D has the capability for large scale biomarker discovery with high performance and accuracy and as examples, Mass++/AB3D algorithms were successfully applied in real biomarker researches [25,26].

## Conclusions

A simple and faster quantitative algorithm called AB3D for large-scale data analysis has been developed as a plug-in for Mass++. The comparison analysis demonstrated that AB3D could properly identify and quantify known peptides with higher true positives and lower false positives comparing with the 4 other previously existing software tools using either the standard peptide mixture or the real complex biological samples of *Bartonella quintana (*strain JK31). Furthermore, AB3D demonstrated the best reliability by comparing the variability between two technical replicates using a complex peptide mixture of HeLa and BSA biological samples. For performance, the AB3D algorithm is about 1.2- 15 times faster than those of existing 4 software tools such as MZmine 2, MSight, SuperHirn and OpenMS. AB3D is very easy to operate with only 3 clicks. In summary, AB3D makes it easier to analyse a large amount of MS data sets with high performance, and provides more reliable information for researchers.

Currently, AB3D is implemented as one of the quantitation plug-ins in Mass++, which is universal free software for mass spectrometric data (available at http://www.first-ms3d.jp/english/). As one of the plug-ins in Mass++, the source code for AB3D is not publicly opened in accordance to the policy of the Mass++ software.

Finally, the authors want to emphasize here that to carry out large scale biological data analysis, sophisticated chromatographic alignment algorithms which were reported and/or to be newly developed are also another key point, currently AB3D employs the chromatographic alignment algorithms developed in the Mass++ software for large scale analysis, other types of chromatographic alignment algorithms will be integrated in future work.

## Methods

### Sample preparations and mass spectrometric analysis

Data set 1:

Bovine serum albumin digestion standard (Michrom Bioresources) was serially diluted and mixed with the tryptic digest standards of the four proteins beta galactosidase, phosphorylase b, myoglobin and cytochrome c, which were purchased from Proteabio Biosciences, to prepare peptide mixture samples. Final peptide concentrations of serially diluted BSA digestion standard were 96, 480, 2400 amol, 12, 60, 300 fmol and that of four proteins were 75 fmol. LC-MS analysis of each peptide sample was performed using a LC-20 AD nano LC pump (Shimadzu), a HTC-PAL autosampler (CTC Analytics) and a LTQ-orbitrap (Thermo Fisher Scientific) with an in-house-built nano-sprayer (100 um inner diameter, 150 mm length) packed with ReproSil-Pur C18 materials (3 um, Dr. Maish). Four LC-MS runs were performed for each prepared sample. The mass spectrometer was operated in the data-dependent mode to automatically switch between MS full scan and MS2 at a spray voltage of 2200 V. The MS scan range was *m/z* 300–1500, and the top five precursor ions were selected from the MS scan for subsequent MS/MS scans by ion trapping. The normalized Collision-induced dissociation (CID) was set to at 35.0. The mobile phases for ODS separation at LC-MS consisted of (A) 0.5% acetic acid in 4% acetonitrile and (B) 0.5% acetic acid in 80% acetonitrile. The gradient was 0% B (0-5 min), 0-37% B (5-20 min), 37-68% B (20-25 min), 68-100% B (25-26 min) and 0% B (26-60 min) at a flow rate of 500 nl/min.

Data set 2:

To test our algorithm can adapt for the real experiment data, a complex biological data set (data set 2) was obtained from the PRIDE database (http://www.ebi.ac.uk/pride/archive/projects/PXD000076). The data set identifier is PXD000076 in the PRIDE database, and the identification results (pmic7388-sup-0001-S1.zip) for data set 2 were obtained from the publisher’s web site (http://onlinelibrary.wiley.com/doi/10.1002/pmic.201200165/suppinfo/). The *m/z* and RT information for each identified peptide were obtained from the .dat files and peptide information of the identification results in the literature, and then comparisons were carried out between peaks detected using the five algorithms and the values reported in the literature. The *m/z* and the RT tolerance were set to 100 ppm and 1 minute, respectively. If the *m/z* and RT values of detected peaks matched the corresponding values reported in the literature within the *m/z* and RT tolerance, we set the detected peak to TP for all five algorithms.

Data set 3:

To assess the reliability of each algorithm with complex peptide samples, HeLa cell proteins digested peptides were prepared by phase transfer surfactants (PTS) method following the previous article [27]. Peptides derived from 75 ng of HeLa cell proteins were mixed with 96 amol of BSA digestion standard and measured by LC-MS under the same conditions as described in Data set 1 earlier. The standard peptides of BSA were utilized for estimation of retention time drift between two replicates and conditions (protein database, precursor and MS/MS tolerances, enzyme, missed cleavages) for BSA standard peptides identification are the same as described in the next section for data set 1.

### Peptide and protein identification for data set 1

The data acquired from LC-MS were searched using Mascot (Matrix Science) and X!Tandem (The Global Proteome Machine Organization) against an in-house built local database consisting of BSA and four standard proteins (beta galactosidase, phosphorylase b, myoglobin and cytochrome c), sequences for each standard protein in the local database were downloaded from UniProt (release 2014_03). The precursor ion mass tolerance was set at ±10 ppm, the MS/MS tolerance was set at ±0.8 Da, the False discovery rate (FDR) was set to <0.05 by decoy search and trypsin was designated as the proteolytic enzyme with 2 missed cleavages. To identify as many peptides as possible with considering different types of post-translational modifications(PTMs), we conducted MS/MS searches against five protein database with consideration in total of 119 and 132 PTMs for Mascot and X!Tandem, respectively. The details about PTMs were provided in Additional file 2. Identified peptides were obtained from the BSA digested standard and the other four digested protein standards such as beta galactosidase, phosphorylase b, myoglobin and cytochrome c peptides. The criteria for identified peptides were as follows: if a peptide score larger than 25 for Mascot or the expectation value <0.1 for X!Tandem and identified from multiple samples for all BSA peptide concentrations, then the peptide was defined as TP in this study. Finally, the numbers of identified peptides for BSA, beta galactosidase, phosphorylase b, myoglobin and cytochrome c peptides were 210, 50, 34, 11 and 11 respectively. In addition, the sequence coverage of these peptides for BSA, beta galactosidase, phosphorylase b, myoglobin and cytochrome c were 87.15%, 34.8%, 32.3%, 50.98%, and 60.58% , respectively (Table 3).

### Software comparison

Parameter settings for all software including a simple operational procedure for AB3D/Mass++, are provided in Additional file 1 and Additional file 4 for data sets 1 and 2, respectively.

### Availability of supporting data

The data sets supporting the results of this article have been deposited to the ProteomeXchange with identifier PXD001259.

## Additional files

Additional file 1:
**Parameter settings of each algorithm for data set 1 and 3 and the operational procedure for AB3D/Mass++.**
Additional file 2:
**PTMs parameters and all identification results using Mascot and X!Tandem for data set 1.**
Additional file 3:
**Features detected from five algorithms for data set 1.**
Additional file 4:
**The tuning parameters of each algorithm for data set 2.**
Additional file 5:
**Features detected from five algorithms and the aligned results between two replicates for data set 3.**

## Abbreviations

AB3D: A simple label-free quantitation algorithm for Biomarker Discovery in Diagnostics and Drug discovery using LC-MS
MOA: Mode of action
XIC: Extracted ion chromatogram, RT, Retention time
*m/z*: mass-to-charge ratio
BSA: Bovine serum albumin
API: Application Programming Interface
S/N: Signal to noise
FWHM: Full width at half maximum
FP: False positive
TP: True positive
FPR: False positive rate
TPR: True positive rate
CID: Collision-induced dissociation
